# Effects of Seed Predators of Different Body Size on Seed Mortality in Bornean Logged Forest

**DOI:** 10.1371/journal.pone.0011651

**Published:** 2010-07-19

**Authors:** Yann Hautier, Philippe Saner, Christopher Philipson, Robert Bagchi, Robert C. Ong, Andy Hector

**Affiliations:** 1 Institute of Evolutionary Biology and Environmental Studies, University of Zurich, Zurich, Switzerland; 2 Sabah Forestry Department, Forest Research Centre, Forestry Department Sabah, Sandakan, Sabah, Malaysia; Centre National de la Recherche Scientifique, France

## Abstract

**Background:**

The Janzen-Connell hypothesis proposes that seed and seedling enemies play a major role in maintaining high levels of tree diversity in tropical forests. However, human disturbance may alter guilds of seed predators including their body size distribution. These changes have the potential to affect seedling survival in logged forest and may alter forest composition and diversity.

**Methodology/Principal Findings:**

We manipulated seed density in plots beneath con- and heterospecific adult trees within a logged forest and excluded vertebrate predators of different body sizes using cages. We show that small and large-bodied predators differed in their effect on con- and heterospecific seedling mortality. In combination small and large-bodied predators dramatically decreased both con- and heterospecific seedling survival. In contrast, when larger-bodied predators were excluded small-bodied predators reduced conspecific seed survival leaving seeds coming from the distant tree of a different species.

**Conclusions/Significance:**

Our results suggest that seed survival is affected differently by vertebrate predators according to their body size. Therefore, changes in the body size structure of the seed predator community in logged forests may change patterns of seed mortality and potentially affect recruitment and community composition.

## Introduction

Human-induced changes to tropical ecosystems are manifold and a major threat to biodiversity. Currently, less than half of the original forests of South-East Asia remains and the levels of biodiversity are predicted to decrease by 42% during this century [1]. In the state of Sabah, Malaysia, our study area, local wildlife populations are depressed by hunting and are becoming depleted or extinct [2]. Understanding to what extent such changes to natural wildlife populations may affect forest dynamics with regard to seed dispersal and seedling survival is an important requirement for the management of tropical forests.

In South-East Asia the Dipterocarpaceae represent 80–90% of the upper canopy of intact lowland forests [3], [4]. The impact of changes to natural wildlife populations on Dipterocarps may have particularly important consequences for the dynamics of Southeast Asian lowland forests. Dipterocarps have evolved a reproductive strategy of interspecific synchronized seed production (mast-fruiting) once every several years interspersed by irregular periods of low seed production [5]. However, although mast-fruiting has been hypothesized as an evolutionary response that allows for the survival of seeds by satiating predators [6], dipterocarp seedling recruitment failure has been reported in Indonesian Borneo following mast fruiting [7] and recent studies in Sabah recorded Dipterocarp seed survivals in non-mast years (R. Bagchi, C. Philipson, unpublished data).

Dipterocarp vertebrate predators in Bornean forests range from the large-bodied bearded pig (57–83 kg) [8], [9] to small rodents (<400 g) [10], [11]. Logging and hunting in tropical rain forests cause changes in small and large vertebrate predator densities, movements and assemblages [9], [11] that may modify natural enemy effects on dipterocarp seedling survival. For example, small-bodied predators are less prone to hunting, they exhibit high population fluctuations that may influence colonization and compensation for local extinction [12]. Conversely, although large-bodied consumers of dipterocarp seeds can increase dramatically during mast years due to influx from surrounding degraded or agricultural areas [13] they are generally more abundant in remote or well protected areas rather than in fragmented forests that facilitate easy access by hunters. Consequently, the decrease in large predator populations due to logging and hunting during non-masting years implies that large predator effects on tree survival may decrease in relative importance.

Several hypotheses have been suggested to account for the maintenance of high levels of tree diversity in tropical forests [14]. Of these, the Janzen-Connell hypothesis is the most widely accepted although its importance as a coexistence mechanism has not been clearly demonstrated [15]–[17]. Janzen [18] and Connell [19] argue that species-specific predators concentrate their activities near adult trees where seed density is high (density effect) and cause higher mortality of seeds and seedlings near the maternal parent (species identity effect) than further away (distance effect). Reduced seed and seedling survival near conspecific adults inhibits the regeneration of abundant species, favours rare species survival and increases the probability of heterospecific establishment. This mechanism limits the potential of single species dominance in the community and could be a significant force in maintaining the high diversity of trees in tropical communities. Seeds and seedlings represent the most vulnerable stage in a tree's development [20]–[22] and density and distance-dependent processes are thought to occur most strongly during these early stages when individuals are most abundant and susceptible to higher mortality [23], [24].

**Figure 1:**
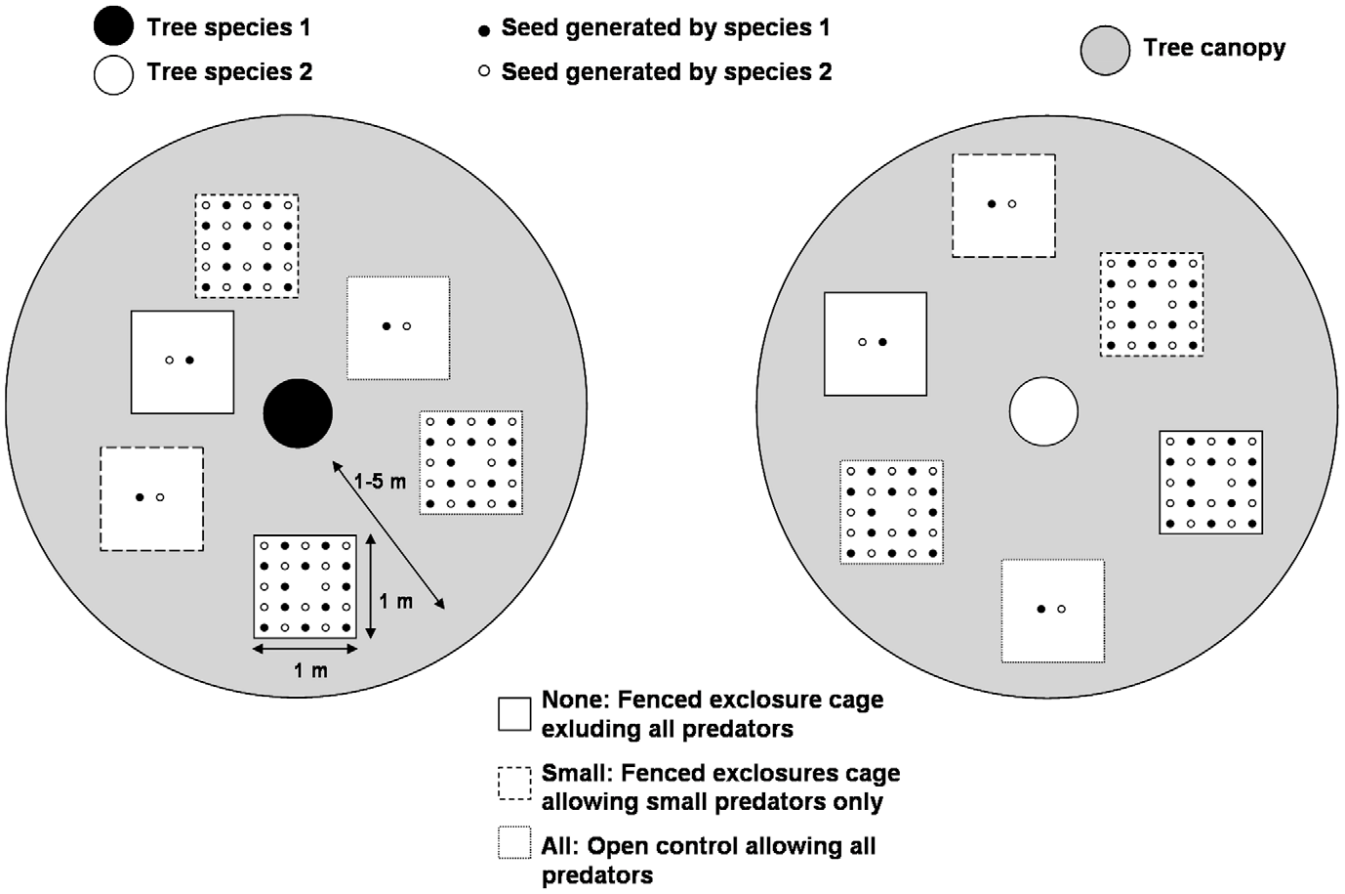
Experimental design. Seeds of each pair of trees (5 pairs) were placed between 1 and 5 m around each tree at high density (24 seeds, 12 seeds from the maternal tree (conspecific) and 12 seeds from a distant tree of a different species (heterospecific) and at low density (2 seeds, 1 con- and 1 hetero-specific). The experimental design consists in three exclosure treatments (1×1 m large ×0.5 m tall): (1) None, fenced exclosure cage excluding both large and small vertebrate predators, (2) Small, fenced exclosure cage excluding only large vertebrate predators and (3) All, open control allowing both small and large vertebrate predators.

Because the effects predicted by the Janzen-Connell hypothesis may depend on the size of predators and on their potential contributions to seed mortality [25], the differences in responses of small versus large predators to logging and hunting have the potential to affect seedling survival in fundamental ways and ultimately influence dipterocarp composition and diversity. Vertebrate effects on dipterocarp seedling survival may change in relative importance with changes in the abundance of predators of different body sizes [12], [26]. Indeed, because small and large vertebrate predators have unique attributes (resource preference, home range, behaviour, population dynamics and community structure) effects predicted by the Janzen-Connell hypothesis may depend on the size of vertebrate predators and on their potential contributions to seed mortality [25]. In this study we investigated the separate contributions of small and large vertebrate predators to seed survival at high and low seed density in logged forest by comparing survival from uncaged control with cages that excluded large predators or both small and large predators (see Fig. 1). We note that while this study focuses solely on predation by vertebrates, mortality due to insects, fungi, bacteria and viruses have all been cited as important drivers of Janzen-Connell effects [16]–[19], [23]. Several insect seed predators of dipterocarps have been identified and may have important effects on dipterocarp seed and seedling survival [27]–[29]. We focused on the seed-to-seedling transition. We show that small-bodied predators selectively predated seeds of the maternal tree, an effect that was cancelled out when large-bodied predators had access to the seeds.

## Results

**Figure 2:**
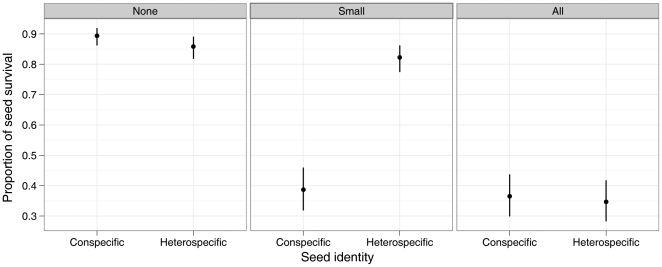
Predator size class effect on con/hetero-specific seed survival. Percentage of mean seed survival in exclosures that allowed vertebrate predators of the specified size classes either close (conspecific) or away (heterospecific) from maternal tree. Results are shown as means ± s.e.m. back transformed from the generalized linear mixed-effects model analysis.

The analysis of seed survival showed strong support for an interactive effect of seed predator body size (exclosure) with the identity of the seed (con- or heterospecific) (Fig. 2; models without the interaction had ΔAIC*_c_* ≥31.6, Table 1). In exclosures from which all the vertebrate predators were excluded (None; Fig. 2) seed survival was relatively high and comparable between conspecific seeds (seeds from the maternal tree) and heterospecific seeds (seeds from the tree of the other species) (Table S1). In contrast, in exclosures open to vertebrate predators, effects on seed survival differed with seed identity. Small predators (Small; Fig. 2) significantly reduced conspecific seed survival from 89.4% (±1 SEM  = 86.2–91.9%) to 38.7% (31.9–46.0%) but did not reduce heterospecific survival (mean ±1 SEM  = 82.3% (77.5–86.2%)) compared with exclosures from which all the predators were excluded (85.8% (81.8–89.1%)). On the other hand, large predators (All; Fig. 2) had no supplementary effect on conspecific seed survival (36.5% (29.9–43.7%)) compared with small predator effects, but significantly reduced heterospecific survival to 34.7% (28.3–41.7%), therefore cancelling out the advantage provided by small predators to heterospecific seed survival. These effects were consistent across the five dipterocarp tree species used in this experiment (Fig. S1).

**Table 1:** Comparison of models with different treatment effects using information-theoretic model selection procedures of candidate models with different fixed effects.

A) Model	AIC*_c_*	ΔAIC*_c_*
A5. seed identity * exclosure	836.7	0
A4. seed identity + exclosure	868.3	31.6
A3. exclosure	880.9	44.2
A2. seed identity	900.1	63.4
A1. intercept	912.8	76.1

We assessed the hypotheses that predator size class (“exclosure”) influences con/hetero-specific (“seed identity”) seed survival differently ΔAIC*_c_* show the change in AIC*_c_* compared to the best model. Models within 2 ΔAIC*_c_* units have equivalent empirical support, those within 4 have a lot of empirical support [52]. The models are ordered according to ΔAIC*_c_*.

**Figure 3:**
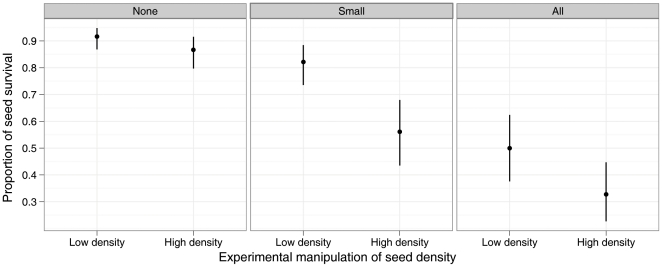
Predator size class effect on seed survival at high vs low experimental seed density. Percentage of mean seed survival in exclosures that allowed vertebrate predators of the specified size classes at the high and the low experimental seed density treatment. Results are shown as means ± s.e.m. back transformed from the generalized linear mixed-effects model analysis.

Model comparison of the effects of predator size class and seed density effect shows that the best model includes an effect of body size of seed predator (exclosure) and an effect of density on seed survival (Fig. 3; Table 2). However, the two models with the next best support (ΔAIC*_c_*<4) include an interactive effect of predator size class and density and an effect of predator size class only, suggesting that we should not rule out an interaction. According to our a priori hypothesis we performed the orthogonal contrasts on the model with interaction. In exclosures from which all the vertebrate predators were excluded (None; Fig. 3) seed survival was relatively high and comparable between low and high seed density (Table S2). Small predators (Small; Fig. 3) did not reduce seed survival at low density (mean ±1 SEM  = 82.2% (73.6–88.4%)) compared with exclosures from which all the predators were excluded (91.6% (86.8–94.8%)) however large predators (All; Fig. 3) significantly reduced seed survival at low density (to 50.0% (37.6–62.4%)). On the other hand, small predators significantly reduced seed survival at high seed density (56.1% (43.5–67.9%)) compared with exclosures from which all the predators were excluded (86.6% (79.7–91.5%)) and large predators further reduced seed survival at high density to 32.8% (22.7–44.7%). This reduction of seed survival at high density by small predators and at both high and low density by large predators was consistent across the dipterocarp tree species used in this experiment, with the exception of *Dipterocarpus caudiferus* (Fig. S2).

**Table 2:** Comparison of models with different treatment effects using information-theoretic model selection procedures of candidate models with different fixed effects.

B) Model	AIC*_c_*	ΔAIC*_c_*
B4. density + exclosure	877.1	0
B5. density * exclosure	880.5	3.4
B3. exclosure	880.9	3.8
B2. density	911.1	33.9
B1. intercept	912.8	35.7

We assessed the hypotheses that predator size class (“exclosure”) influences seed survival differently at high vs low experimental seed density treatment (“density”). ΔAIC*_c_* show the change in AIC*_c_* compared to the best model. Models within 2 ΔAIC*_c_* units have equivalent empirical support, those within 4 have a lot of empirical support [52]. The models are ordered according to ΔAIC*_c_*.

## Discussion

Logging and hunting pressures in tropical rain forests are leading to reduced populations of large seed predators. As the mechanisms maintaining dipterocarp diversity in logged forest areas may depend on the size of seed predators [26], [30], the decrease in large seed predator populations may affect their impact on dipterocarp survival.

To assess the species-specific effects of seed predators predicted by the Janzen-Connell hypothesis (higher seed mortality by seed predators close to the maternal tree) we swapped seeds from pairs of trees belonging to different species while excluding seed predators of different body size. We generally found that small seed predators decreased seed survival of seeds from the mother tree (conspecific seeds) while large seed predators reduced heterospecific seed survival. We also assessed the density effect predicted by Janzen-Connell through experimental manipulation of seed density (experimental seed density treatment). We found that small predators reduced seed survival at high seed density while large predators reduced seed survival at both high and low seed density.

Small seed predators are usually ubiquitous and occupy small territories that they rigorously inspect [31]. They may prefer local seeds and be able to discriminate between seed species based on their size [12], [32]–[34]. This behaviour may explain the result observed: when small seed predators were allowed access they consumed disproportionately more seeds from the maternal tree, leaving seeds coming from the distant tree of a different species. The low predation rate at low seed density could be explained as follow. In addition to the seeds experimentally placed around the trees, the number of background seeds collected under the tree canopies during the experiment ranged between 118 and 2286 (Table 3). Small seed predators might have initially concentrated on the high seed density treatment and on the background seeds. These may have satiated predators before they could exploit the less-profitable, low-density plots. Interestingly, at one of the sites we observed that a fiddler crab had carried seeds into its hole and this species may be a previously undiscovered disperser and predator of dipterocarp seeds (Fig. S3). In the Neotropics, crabs have been recently shown to be important for local dispersal of freshly fallen seeds [35].

**Table 3:** Characteristics of the study trees.

Tree pair	Tree species	DBH* (cm)	Number of seeds collected between the 10^th^ and the 20^th^ of February 2007	Mean seed biomass (g)	Distance between the pairs of tree (m)
1	*Parashorea tomentella* (Sym.)	240	284	4.0	589
1	*Shorea leprosula* (Miq.)	200	903	0.6	
2	*Shorea parvistipulata* (Heim.)	216	118	1.8	558
2	*Shorea johorensis* (Foxw.)	370	855	0.9	
3	*Shorea johorensis*	250	2286	1.0	878
3	*Shorea leprosula*	169	858	0.7	
4	*Dipterocarpus caudiferus* (Merr.)	225	126	6.5	798
4	*Shorea leprosula*	174	135	0.7	
5	*Shorea parvistipulata*	307	461	1.3	521
5	*Shorea leprosula*	184	775	0.6	

*DBH: diameter at breast height.

Unlike small seed predators, larger species occupy large home ranges where they may travel long distances to feed on high density of resources [8], [27]. This behaviour could explain why seed survival was greatly reduced when all predators had access independently of the seed species identity and density.

The background seed density may interfere with the experimental seed density treatment to affect seed survival; a higher background seed density may attract more predators and therefore inflict lower survival independently of the experimental seed density treatment. In our study adding the background seed density as a covariate did improve the model slightly (ΔAIC*_c_* = 0.97) but the qualitative results remains very similar. This suggests that the results of our experimental seed density treatment were not influenced by the background seed density. Similarly, seeds with higher biomass may be more attractive or simply more visible and predators could inflict lower survival independently of the con/hetero-specific treatment. In our study adding seed biomass as a covariate had limited direct effects on seed survival (ΔAIC*_c_* = 5.6). Large seed predators reduced survival of large-seeded species (slope with 95% CI on the logit scale  = −0.30 (−0.51 – −0.08)) whereas there was no relationship between seed removal and seed biomass in exclosures open to small seed predators (slope with 95% CI on the logit scale  = −0.12 (−0.29–0.04)) or in exclosures from which all the predators were excluded (slope with 95% CI on the logit scale  = −0.14 (−0.33–0.10)).

The Janzen-Connell effect predicts high seed mortality by seed predators close to the maternal tree where seed density is high. Our results show that small seed predators caused disproportionately higher mortality of seeds from the maternal tree, potentially generating an advantage for establishment of seeds from other dipterocarp species. Furthermore, they appear to prefer areas of high density, suggesting that they might be optimising their foraging on a local scale. This process could, as suggested by the Janzen-Connell hypothesis, promote diversity at both the landscape and local scales. On the other hand, predation by large vertebrates appears to be independent of seed identity and density but related to seed biomass, at least at the scale examined.

Although density and distance effects on seed and seedling survival have been widely studied in the tropics [15,23,reviewed in 36,37,38] and in dipterocarps [7], [39]–[41], no consensus has been reached over the general importance of the Janzen-Connell effect. A meta-analysis based on 40 papers and 75 species reported that even though distance-dependent survival is evident for some species, the data available did not support the Janzen-Connell hypothesis to be a general phenomenon across communities [15]. A compilation of theoretical and experimental studies suggests that invertebrates support the predictions of the Janzen-Connell model, but vertebrates do not [23]. By separating the individual effects of small and large seed predators our results may explain why outcomes of previous experiments testing the Janzen-Connell hypothesis have been so disparate. Varying results from studies of the Janzen-Connell effect might arise in part from the structure of the seed predator community and whether it has been affected by human activities (logging, hunting, etc) [42]–[44]. Due to their small territory occupation and more focused search image small seed predators can create an advantage for heterospecific seedling survival success which is the key of the Janzen-Connell hypothesis. However, if the effect of small seed predators is confounded with that of large seed predators, as is usually the case in natural ecosystems, the evidence for distance-dependence may disappear.

## Material and Methods

### Study site and selection of trees

This study was conducted during a partial fruiting event that occurred in February 2007 close to Taliwas, 25 km west of Lahad Datu, on the east coast of Sabah, Malaysian Borneo (4°58′ N, 118°06′ E). The site was located in an alluvial plain and covers an area of 5.4 ha. This area experiences a wet equatorial climate. Temperature and precipitation are comparable to the Danum Valley Field Centre [8], [45] where mean annual temperature is 26.7°C, mean maximum temperature is 30.9°C and mean minimum is 22.5°C. Average rainfall is about 2700 mm per year, although severe droughts regularly occur influenced by the El Niño Southern Oscillation (ENSO) events that were recorded during 1986–87, 1991–94 and 1997–98 and 2010. This lowland tropical secondary forest was logged in the early 1970s [45] and is still dominated by trees from the family Dipterocarpaceae. Dipterocarps produce fleshy single-seeded fruits that are dispersed by wind, water and animals. The seeds are recalcitrant, meaning that they germinate within days of dispersal and display no seed bank [46].

In the beginning of February 2007, we selected ten easily accessible fruiting adult trees (169–370 cm DBH) belonging to five species of Dipterocarpaceae: *Parashorea tomentella, Shorea leprosula, S. parvistipulata, S. johorensis* and *Dipterocarpus caudiferus* (Table 3). Trees were situated between 138 m and 182 m above sea level and between 21 m and 139 m from the logging road (see Table 3 for a complete description of the trees). All trees were located in logged forest except one individual of *S. leprosula* that occurred in a *Nephelium lappaceum* L. (Rambutan) plantation.

### Experimental design

Three types of exclosures were designed to exclude the access of small or large vertebrate predators. Small-bodied consumers of dipterocarp seeds in Bornean forests include squirrels (*Ratufa affinis*, *Sundasciurus hippurus, Callosciurus adamsi*, *C. prevosti* and *C. notatus*) and rodents (*Maxomys surifer, M. rajah, M. whiteheadi, Leopoldamys sabanus* and *Sundamys muelleri*) [13], [25], [47]–[49] and large-bodied consumers of dipterocarp seeds include pigs (*Sus barbatus*) and macaques (*Macaca fascicularis* and *M. nemestrina*). The name of each exclosure indicates the size class of vertebrates that were permitted access. Small and large vertebrates were excluded (None) using wire-mesh cages (1×1 m, 0.5 m tall and 1 cm mesh size). The effect of small vertebrate predators was isolated using identical exclosure cages to None but with four openings along each side of the cage (10×10 cm) that excluded large vertebrate predators allowing only small vertebrate predators access (Small). Small and large vertebrate predators were allowed to access an open control (All). Exclosures were secured at their base with spikes so that the mesh was tight against the ground. This experiment was an incomplete factorial design because it was not feasible to exclude small vertebrate predators and not exclude large vertebrate predators at the same time.

Freshly fallen seeds were collected from under the canopy of the selected trees one to two days before placing in the cages. Seeds with indications of predation, fungal damage or germination were discarded. The biomass of the seeds used in the experiment was predicted using regressions relationships established for each species from 20–50 additional seeds dried at 80°C to constant mass. The average seed weight of species used in this experiment ranged from 0.7–6.5 g (Table 3).

The Janzen-Connell effect predicts high seed mortality by specialist seed predators close to the maternal tree where seed density is high. Therefore, to estimate both the con/hetero-specific and density effects we swapped seeds from pairs of trees belonging to different species into high and low density treatments. The con/hetero-specific effect inevitably includes a distance effect. Trees were paired in a way that minimised the difference in the distance between the five pairs of trees (between 521 and 878 m). To estimate the con/hetero-specific effect, for each tree in the pair seeds coming from the conspecific tree (seeds belonging to the maternal tree of the pair) and from the heterospecific tree (seeds belonging to the distant paired tree of a different species) were placed within each exclosure at the same density of seeds (crossed experimental design, see Fig. 1). To estimate the density effect, for each exclosure type seeds were placed at either high density (corresponding to natural density: 24 seeds/m^2^, 12 con- and 12 hetero- specific) or low density (2 seeds/m^2^, 1 con- and 1 heterospecific) [30]. This density treatment (experimental manipulation of seed density) is called hereafter “experimental seed density treatment” to distinguish it from background seed density (below). Seeds were placed 15 cm apart (see Fig. 1). We followed the fate of the seeds by tethering each seed with a 3 m string to a nail dug into the soil at the position of the seed. Exclosures were placed randomly within a radius of 1–5 m randomly from the trees.

Background seed densities under the canopy of the parent tree (unmanipulated seed densities), hereafter “background seed density”, were determined from seeds that fell on the experimental cages (four cages of 1×1 m per tree) and in three litter traps of 1×1 m placed at breast height and set up randomly at 2, 3 and 4 m from each tree. Moreover, to prevent large differences in the background seed densities between the selected trees interacting with the experimental seed density treatment, every three days during the period of the experiment freshly fallen seeds were collected on the soil below the tree canopy where the density was highest. To assess the influence of the background seed densities on the experimental seed density treatment we estimated the effect of the background seed density on seed predation (see *Statistical analysis* section below).

Seeds were monitored on day 18 (the majority of dipterocarp seeds germinate within days of dispersal [50]) and scored as alive, germinated or missing. We used removal as an indicator for seed predation assuming that seeds found missing were either immediately eaten at tethered locations or some time later if they were cached by rodents [34]. Hence, missing seeds, gravely damaged seeds and seeds that did not germinate were scored as dead while germinated seeds that were intact or slightly damaged were scored as alive.

### Statistical analysis

We used generalized linear mixed-effects models (GLMMs) [51]–[55], with a binomial error distribution since our design includes fixed and random effects and seed mortality is a binary response variable. The GLMMs were fitted using restricted maximum likelihood (REML) with the lmer function from the lme4 library [52] for R 2.8.1 [56].

Our focal interests in this analysis regarded the effects of each predator size class on the con/hetero-specific and density effects predicted by the Janzen-Connell hypothesis. Therefore, we followed the practical guide for GLMMs advocated by Bolker *et al.* [55] and compared the goodness of fit among candidate models with different fixed effects and their interactions. Specifically, we tested the hypotheses that predator size class (“exclosure”) influences con/hetero-specific (“seed identity”) seed survival differently and that predator size class (“exclosure”) influences seed survival differently at high vs low experimental seed density treatment (“density”). We used information-theoretic model selection procedures that allow comparison of multiple, nonnested models. The Akaike information criterion (AIC) and related information criteria (IC) use deviance as a measure of fit, adding a term to penalize more complex models (i.e. greater numbers of parameters). Rather than estimating p values, information-theoretic methods estimate statistics that quantify the magnitude of difference between models in expected predictive power (ΔAIC). Our comparison of models with different treatment effects showed that most of the models had ΔAICs higher than 30, these models are implausible and can therefore be dismissed [57]. However two models showed ΔAIC<4, these models have a lot of empirical support and should not be eliminated from scientific consideration. We tested for overdispersion and used a variant of the AIC for small sample size (AIC*_c_*). Using a mixed-effects model approach permitted us to take into consideration the hierarchy of the experimental design with its multiple error terms. The interaction between exclosure and seed identity occurred at the plot level (number of exclosure cages N = 60) and the interaction between exclosure and density occurred at the residual level (number of experimental seeds N = 780). We used a priori orthogonal contrasts to test the separate effects of small and large vertebrates on seedling survival and the impact of each predator size class was determined by contrasting seed survival between exclosure treatments that differed only in their permeability to that size class. Accordingly, we contrasted None vs. Small exclosures to estimate the effect of small vertebrates and Small vs. Large for large vertebrates.

Because the background seed density may interfere with the experimental seed density treatment, we assessed the influence of the background seed densities on seed survival by adding the background seed density as a covariate with the predator size class (“exclosure”) and the seed density (“density”) treatment. Similarly, as seed biomass may interfere with the experimental con/hetero-specific treatment, we assessed the influence of seed biomass on seed survival by adding the seed biomass as a covariate with the predator size class (“exclosure”) and the con/hetero-specific (“seed identity”) treatments.

Tree species and plots were treated as random effects. In the text and figure, we present point estimates of the means from the GLMMs with their standard errors and the slopes with their 95% confidence intervals.

## Supporting Information

Figure S1 Tree specific response to con/hetero-specific seed survival. Percentage of mean seed survival in exclosures that allowed vertebrate predators of the specified size classes either close (conspecific) or away (heterospecific) from maternal tree. Results are shown as means ± s.e.m. back transformed from the generalized linear mixed-effects model analysis for the five dipterocarp tree species used in this experiment.
(9.89 MB TIF)

Figure S2 Tree specific response to seed survival at high vs low experimental seed density. Percentage of mean seed survival in exclosures that allowed vertebrate predators of the specified size classes at the high and the low experimental seed density treatment. Results are shown as means ± s.e.m. back transformed from the generalized linear mixed-effects model analysis for the five dipterocarp tree species used in this experiment.
(9.89 MB TIF)

Figure S3 Fiddler crab (arrowhead) halfway in its hole with the strings and seeds going into the hole. Photo credit: Yann Hautier.
(0.26 MB JPG)

Table S1 Effect of predators of different body size (None, Small, All) on con/hetero-specific seed survival. Results are shown as mean and standard error (logit scale). The effects are reported as the value for the control and the differences (in italics) between control and the other treatments.
(0.04 MB DOC)

Table S2 Effect of predators of different body size (None, Small, All) on seed survival at two experimentally manipulated seed densities (low  = 2 or high  = 24 seeds/m2). Results are shown as mean and standard error (logit scale). The effects are reported as the value for the control (None) and the differences (in italics) between control and the other treatments.
(0.04 MB DOC)
