# Supplementary figures and images for: Effects of Seed Predators of Different Body Size on Seed Mortality in Bornean Logged Forest
Source: PLoS One. 2010 Jul 19;5(7):e11651. doi: 10.1371/journal.pone.0011651 (PMC2906513; doi:10.1371/journal.pone.0011651)

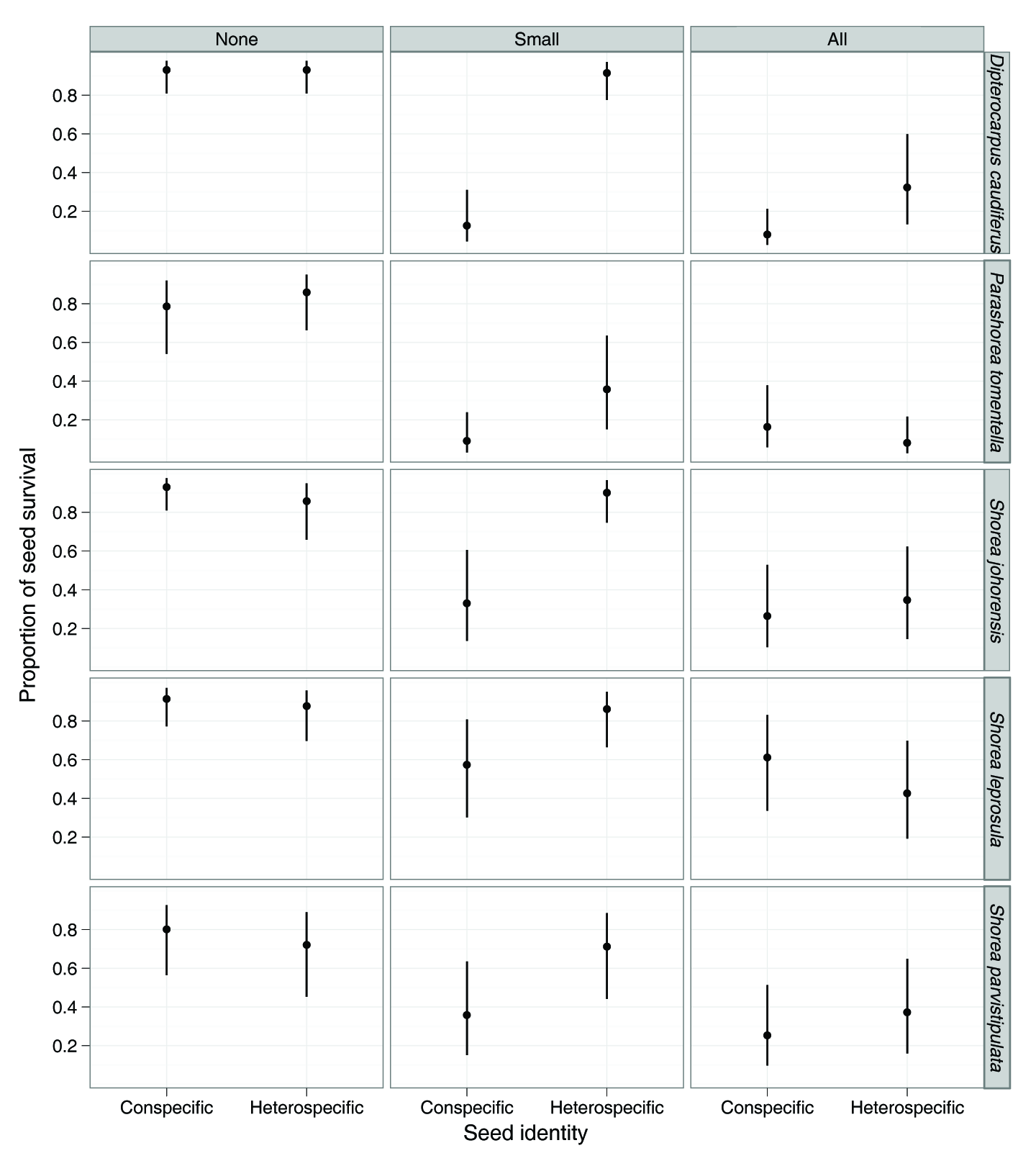

Supplement: Figure S1 — Tree specific response to con/hetero-specific seed survival. Percentage of mean seed survival in exclosures that allowed vertebrate predators of the specified size classes either close (conspecific) or away (heterospecific) from maternal tree. Results are shown as means ± s.e.m. back transformed from the generalized linear mixed-effects model analysis for the five dipterocarp tree species used in this experiment. (9.89 MB TIF) [file pone.0011651.s001.tif]

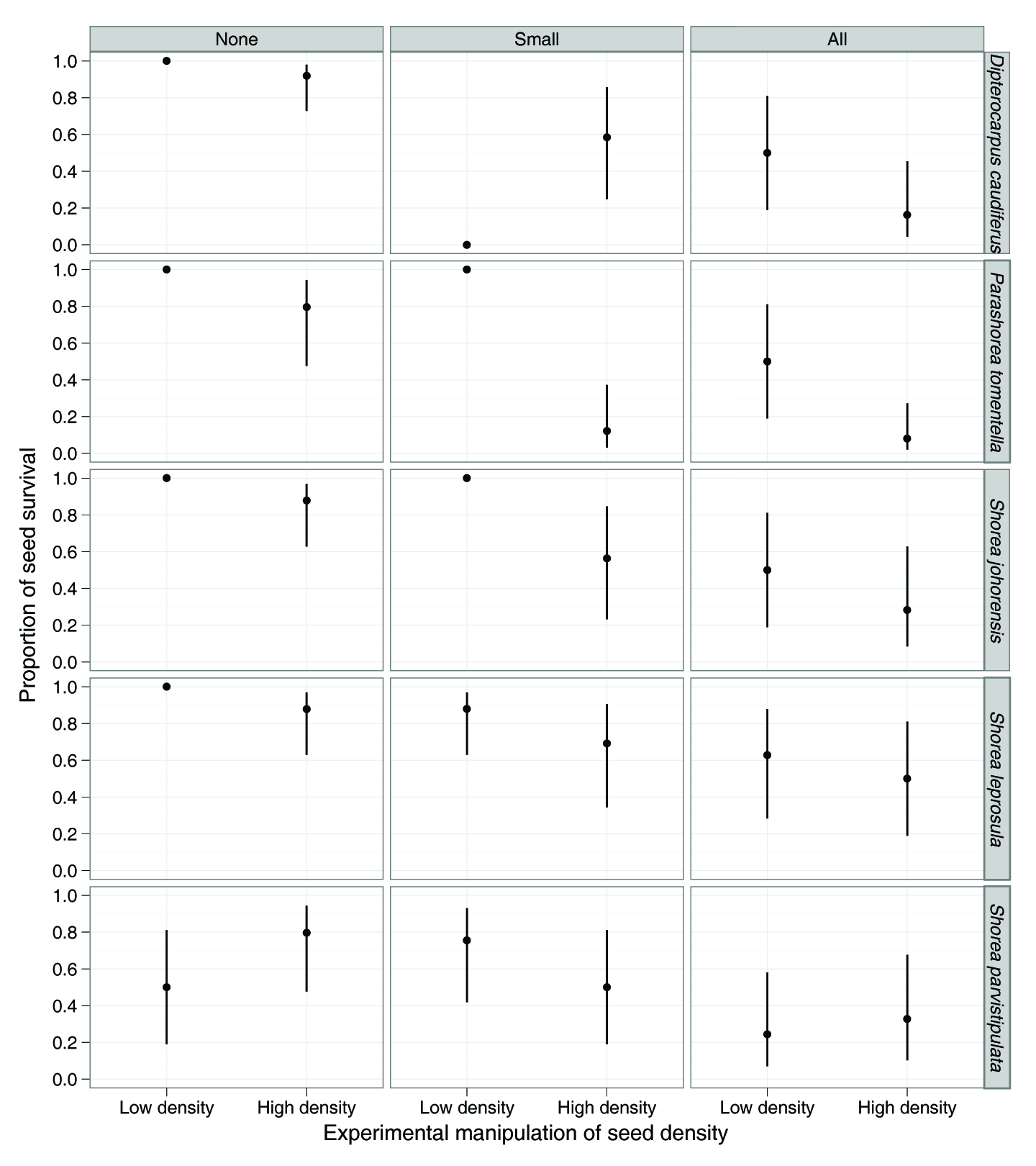

Supplement: Figure S2 — Tree specific response to seed survival at high vs low experimental seed density. Percentage of mean seed survival in exclosures that allowed vertebrate predators of the specified size classes at the high and the low experimental seed density treatment. Results are shown as means ± s.e.m. back transformed from the generalized linear mixed-effects model analysis for the five dipterocarp tree species used in this experiment. (9.89 MB TIF) [file pone.0011651.s002.tif]

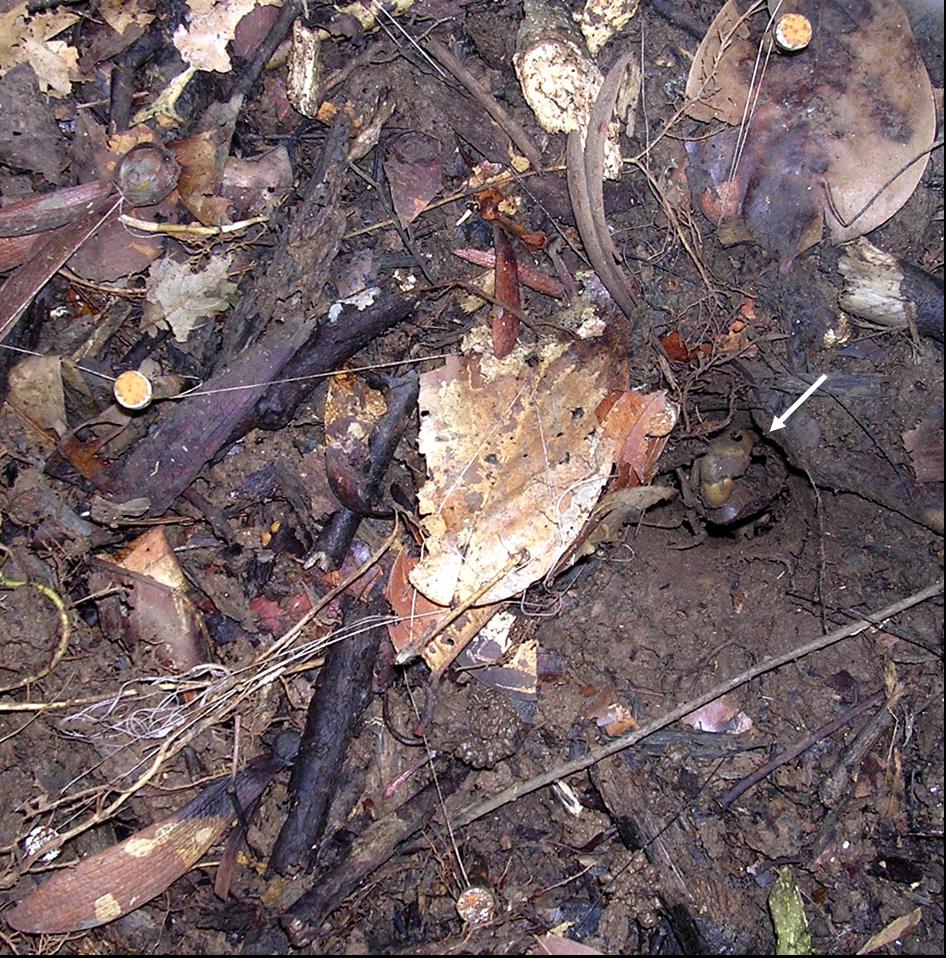

Supplement: Figure S3 — Fiddler crab (arrowhead) halfway in its hole with the strings and seeds going into the hole. Photo credit: Yann Hautier. (0.26 MB JPG) [file pone.0011651.s003.jpg]
