# Supplementary material for: Effects of Seed Predators of Different Body Size on Seed Mortality in Bornean Logged Forest
Source: PLoS One. 2010 Jul 19;5(7):e11651. doi: 10.1371/journal.pone.0011651 (PMC2906513; doi:10.1371/journal.pone.0011651)
Supplement: Table S1 — Effect of predators of different body size (None, Small, All) on con/hetero-specific seed survival. Results are shown as mean and standard error (logit scale). The effects are reported as the value for the control and the differences (in italics) between control and the other treatments. (0.04 MB DOC) [file pone.0011651.s004.doc]

| Source | Estimate | Std. Error | z value | Pr(>|z|) |
| --- | --- | --- | --- | --- |
| None_Conspecific | 2.13 | 0.36 |  |  |
| None_Heterospecific | *-0.33* | *0.37* | *-0.902* | *0.367* |
| Small_Conspecific | *-2.59* | *0.43* | *-6.086* | *<0.001* |
|  |  |  |  |  |
| Source | Estimate | Std. Error | z value | Pr(>|z|) |
| Small_Conspecific | -0.46 | 0.30 |  |  |
| All_Conspecific | *-0.09* | *0.37* | *-0.245* | *0.806* |
|  |  |  |  |  |
| Source | Estimate | Std. Error | z value | Pr(>|z|) |
| None_Heterospecific | 1.80 | 0.34 |  |  |
| Small_Heterospecific | *-0.26* | *0.43* | *-0.621* | *0.534* |
|  |  |  |  |  |
| Source | Estimate | Std. Error | z value | Pr(>|z|) |
| Small_Heterospecific | 1.53 | 0.32 |  |  |
| All_Heterospecific | *-2.17* | *0.40* | *-5.448* | *<0.001* |
